# Ehrlich’s Cure for Syphilis with Hata’s Dioxydiamidoarsenobenzol

**Published:** 1910-09

**Authors:** A. W. Nelson


### Ehrlich's Cure for Syphilis With Hata's Dioxydiamidoarsenobenzol.

Is it possible that at last a specific has been discovered against syphilis? Can we freely accept the present reports which have so stirred up the medical world that one of the greatest plagues that the world has ever known is about to be wiped from the face of the earth? It is almost beyond human credulity to believe that a disease which has in the past destroyed armies, killed men, women and children by the thousands, maimed and scarred by the millions, has at last met its foe in Ehrlich, and is about to be conquered.

During the last few years Ehrlich has devoted himself to the practical experimentation with remedies for the cure of infectious diseases, especially with such agents which are capable of killing all the parasites or rendering them sterile. The accomplishment of this purpose is what Ehrlich calls *sterilisatio magna.* Among the many successful experiments, which he made in a large variety of infections, the most remarkable results were obtained with Hata's diamidoarsenobenzol in cases of syphilis. The manner of administering the remedy consists in the administration of one injection to a syphilitic subject. The use of the remedy in man was preceded by numerous experiments on animals (infection with spirocheta, verification by Wassermann method, administration of the new remedy in the manner indicated above and demonstration of the complete "sterilization" by Wassermann test). The new remedy has been used in a large number and variety of cases by observers in all parts of Germany. There is a singular unanimity of opinion in regard to the therapeutic efficiency of the remedy. Some of the observers speak in terms of greatest praise and enthusiasm of the new method and refer to it as the greatest achievement in biologic therapy. All of the observers agree that the new method represents the greatest progress that has ever been made in the treatment of lues. Perhaps the most remarkable feature of the subject is the uniform adaptability and efficiency of the new remedy in all stages and varieties of syphilis and syphilitic manifestations, from the primary lesion to the ill-defined appearances of an almost forgotten tertiary lesion. The curative effects follow the one injection within ten to fourteen days. The *sterilisatio magna,* which is established within the shortest space of time, is equivalent to a complete disappearance of the spirocheta pallida, and therefore synonymous with a complete cure.

Schneider and Hoppe (*Muenchener Med. Woch.,* July 5, 1910) state that in their series of cases the Wassermann reaction usually was negative in fourteen days after injection of the remedy. The shortest period was four days, while the longest was seventy days.

In all cases the Wassermann test is resorted to to establish the diagnosis before the treatment is begun and to demonstrate the complete *sterilisatio magna* within the short space of time above referred to.

Ehrlich gives the following directions for the intramuscular injections of a 1 to 100 solution of the remedy:

Stir with a glass rod 0.6 g. of the remedy in 3 c.cm. of glycol. The addition of a few drops of water facilitates the solution. Now add 12 c.cm. of water, shake thoroughly and add 10.3 c.cm. n|5 NaOH. The shaking produces a clear solution, to which water is added, until the whole mixture represents 60 c.cm. The whole process should be gone through in a glass graduated measure.

The remedy may be used intravenously. Ehrlich gives the following formula for that purpose: (*a*) Dioxydiamidoarsenobenzol 0.6 gm., methyl alcohol 0.3-0.5 c.cm., or 3 c.c. of glycol. (*b*) Make another solution by taking 240 or more c.c. of physiologic NaCH sol., 10.3 c.c. n|5 NaOH; amid continuous stirring, solutions *a* and *b* are mixed.

The injections are neither accompanied nor followed by any untoward symptoms. In infants the endotoxins, which are liberated during the destruction of the spirocheta, are capable of causing temporary disturbances. The intramuscular injection itself is, however, somewhat painful. The pain is the only unpleasant feature of the treatment, and may last anywhere from several hours to one or six days. The severity of the pain varies according to the sensitiveness of the patient. Schneider and Hoppe usually put their patients to bed for four days even after the disappearance of pain. Frequently the pains are lessened by walking and therefore a difficulty arises in confining such patients to bed. The walking around will occasionally, after three or four days, renew the pains, although it will not be of a severe degree. The same authors have used the intravenous method in thirty cases with no evil results, and claim for it the advantage of freedom from pain and exactness of dosage. In four cases four weeks after intramuscular injections with no results on account of smallness of dose they resorted to the intravenous method with good results. They have found arsenic in the muscles thirty-six days after intramuscular injections of the remedy on account of inflammation, infiltration and walling up of the arsenic. The elevation of temperature, which may run up to 38 degrees C. or even higher, is due to inflammatory reaction. Narcotics may have to be resorted to in cases where the pain is severe.

The usual dose of the remedy is about 0.6 gm. for males and 0.45 gm. for females. It must not be forgotten, however, that there is no more arbitrary dose of this remedy than of any other drug. The dose depends on the patient and the cure depends on sufficient dosage.

Intramuscular injections are made into the gluteal muscles the same as mercury. One-half (30 c.c.) of the remedy is injected on one side, while the remaining half on the other side. Usually an angry looking edema appears at site of injection.

Since mercury has thus far been the sheet anchor in the treatment of syphilis, we feel that reference to W. Wechselmann's cases, as reported before the Berlin Medical Society, June 22, 1910 (*Berliner klinische Wochenschrift,* July 4, 1910), will illustrate the comparative value of the new remedy and of mercury.

CASE I.—Female, 20 years of age, showing universal small papular eruption, mucous patches on the tongue and labia majora covered with papules. An injection of dioxydiamidoarsenobenzol was administered with the following results: Three days after the injection there was distinct involution. Seven days after the injection the papules and mucous patches healed. Return to normal continued and after three weeks all that remained was a little pigmentation in and under the skin.

An almost identical case was subjected to mercurial inunctions for five weeks with practically no effect. An injection of 606 (Ehrlich's dioxydiamidoarsenobenzol) was administered, resulting in marked involution within eight days.

CASE II.—The following case seems almost incredible. Patient, young man, 23 years of age, with a primary lesion seven months ago (November, 1909). Four weeks later there was a general eruption. From November 28, 1909, till the middle of March, 1910, was given thirty-five mercurial injections. The past three months has been having pains in both knee joints. On May 5, the patient was emaciated, skin of a dirty pallor. The face resembled that of a skull, with an almost hippocratic expression; face and body were covered with ulcers, scars and crusts; intensely offensive odor from the nose, septum perforated, part of the nasal bones necrotic with large ulcerations in the posterior nares; uvula almost destroyed. Patient was unable to swallow and has to be fed per rectum. The pulse was small and rapid, 120 and over. In view of the almost inevitable end, 0.4 of Ehrlich's remedy was injected on the 21st of May, which was followed by moderate pain and no elevation of temperature. General condition began to improve after two or three days. On May 26, all ulcerations were healing. On May 30, the remnant of uvula was found healthy and perfectly cicatrized. The diseased nasal bones were expelled *in toto;* offensive odor has disappeared, and on June 7 the patient was able to swallow, can walk about and was constantly gaining in weight.

Many other cases of a similar character have been independently reported by different observers. The reports of the many investigators who have experimented with Ehrlich's new remedy seem to justify the belief that the syphilitic problem has at last been solved, and that Ehrlich's work is an achievement of the first magnitude there is no doubt.

In view of the above reports, it would seem folly for us to do anything else but accept the report of the results as final, and remain satisfied that after many years of investigation a specific for syphilis has been discovered; especially so when the remedy is applicable to all stages of the disease and that cures have been obtained where mercury has entirely failed. Nor will we attempt to deny that the remedy is a specific for syphilis, as such an effort on our part would perhaps stamp us as a tyro or reactionary in medicine, quite contrary to our wishes. It must be admitted, however, that before accepting the remedy as a specific for the permanent cure of syphilis numerous points must be settled. The laboratory and Wassermann test will not settle that either. Time alone will tell. Not sufficient time has elapsed since the various cures have been performed. Practically speaking, the cures reported are only of a three or four months' duration, as Wechselmann began the use of the remedy in April, 1910. Since the cures reported are of recent date, we have no assurance at the present that the cure is permanent. We know that some of the most serious sequelæ of syphilis manifest themselves many years after the infection and apparent cure. At the same time, it must be admitted that before the discovery of the Wassermann test, our conception of a cure of syphilis was vague and empirical and that the Wassermann test helped to settle that point. Yet, in spite of our scientific knowledge, it devolves upon us to be careful before asserting ourselves as to the permanency of a cure resulting from a single injection of a "specific."—*By A. W. Nelson, M. D., in The Lancet-Clinic.*

